# Pulmonary arteriovenous malformation: An uncommon disease with common presentation

**DOI:** 10.4103/0970-2113.71965

**Published:** 2010

**Authors:** Debabani Biswas, Atin Dey, Mukul Chakraborty, Saurabh Biswas

**Affiliations:** *Department of Respiratory Medicine, Calcutta National Medical College, Kolkata, India*

**Keywords:** CT pulmonary angiography, hemoptysis, pulmonary arteriovenous malformation

## Abstract

A 45-year-old male presented with massive hemoptysis, clubbing in all limbs, disproportionate hypoxia and persistent ill-defined shadow in left lower zone in chest radiograph since his childhood. The patient received empirical anti-tuberculosis treatment and the chest X-ray finding was misinterpreted as tuberculoma. Subsequently, CT pulmonary angiography proved it to be a case of a simple type solitary pulmonary arteriovenous malformation with a saccular aneurysm in left lower lobe.

## INTRODUCTION

Pulmonary arteriovenous malformations (PAVMs) are rare abnormalities of the pulmonary vascular system characterized by an abnormal communication between the pulmonary artery and vein, resulting in a low resistant right-to-left shunt. The disease was first described at an autopsy in 1897 and first diagnosed during life in 1939.[1] PAVM was detected in only three cases out of 15000 consecutive autopsies at John Hopkins Hospital.[2] Mayo clinic reported 68[3] and 38[4] cases over a period of more than twenty-five years in two different reports. According to some authors, smaller nontertiary care hospitals might expect to see one case in every few years.[5] Approximately 70% of PAVMs are associated with hereditary hemorrhagic telengectasia (HHT), and about 15-30% of individuals with HHT have a PAVM.[6] Multiple PAVMs are mostly associated with HHT. PAVM may develop later in life in hepatic cirrhosis and hepatopulmonary syndrome, schistosomiasis, mitral stenosis, trauma, actinomycosis, metastatic thyroid carcinoma, and chronic inflammatory condition such as bronchiectasis.[5] Pulmonary arteriovenous malformations occur twice as often in women than in men[7] with male preponderance in newborn.

Herein we report a case of a single, simple type PAVM in an adult male, mostly congenital, considering the rarity of the disease and at the same time emphasizing the resemblance of its radiological findings with frequently encountered chest radiological lesions.

## CASE REPORT

**Figure 1:**
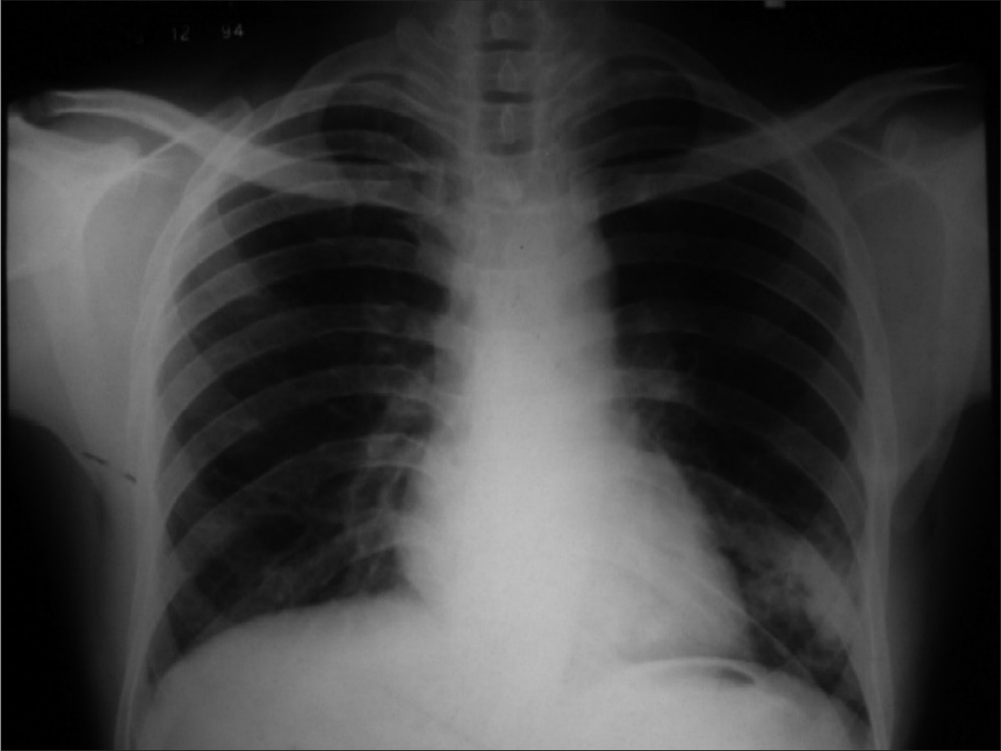
Homogenous opacity in left lower zone in 1994

**Figure 2:**
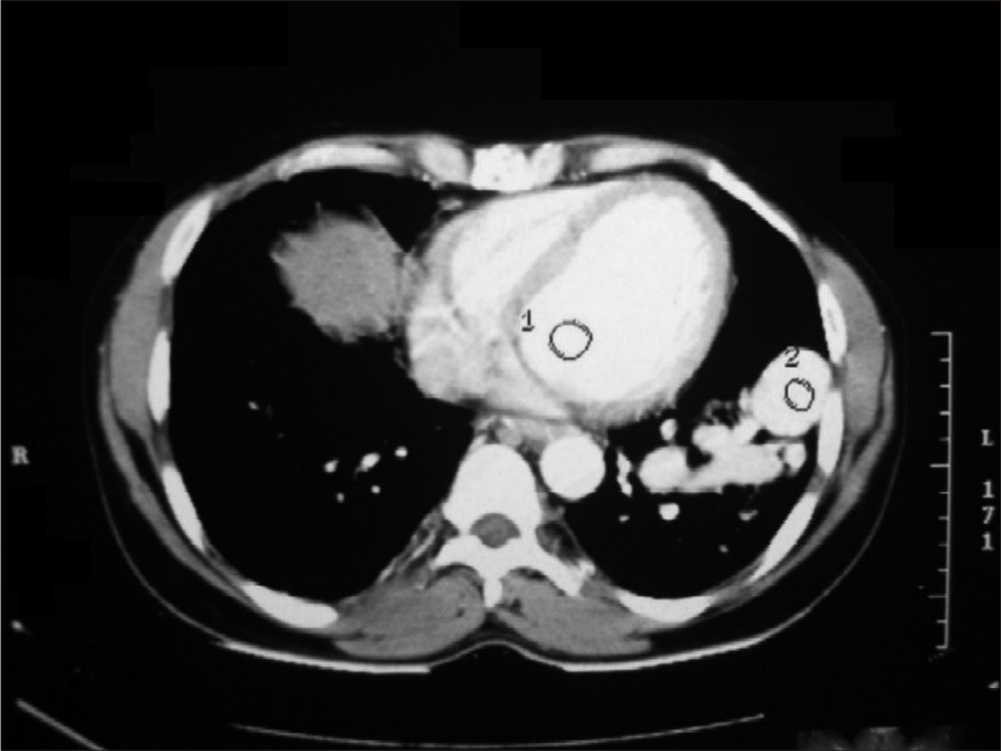
CECT thorax showing abberant vessels in left lower lobe

**Figure 3:**
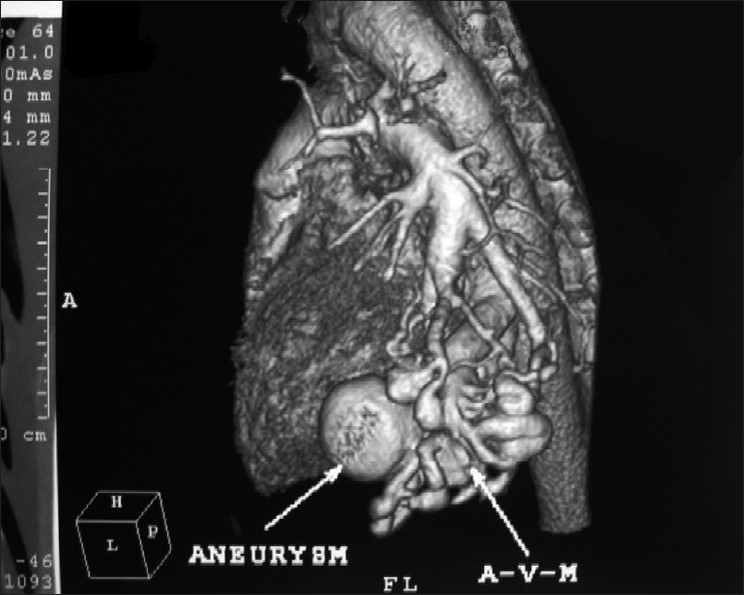
Reconstructed figure showing saccular aneurysm with feeding artery and draining vein

A 45-year-old non-smoker, non-alcoholic Hindu male patient presented to our out-patient department with recurrent scanty hemoptysis over a period of one month and a single bout of massive hemoptysis on the day of admission. He was little short of breath while walking even on level ground when compared to persons of his age and sex. He had no associated history of cough, expectoration, fever, chest pain, chest trauma or surgery in chest. His past history was suggestive of repeated antibiotic treatment for recurrent cough and cold, occasional scanty hemoptysis and an abnormal chest radiological finding since childhood including a course of empirical anti-tuberculosis treatment for one year in his late twenties. The chest radiographic finding persisted even after completion of ATD and was explained as a tuberculoma [Figure 1]. He was apparently healthy for the next fifteen years. None of his family members had similar illness, or any cardiac, hepatic, neurological or dermatological problem. Clinically, patient was afebrile, alert, conscious, tachycardic (120/min regular), tachypnic (28/min) with blood pressure 100/60 and reduced oxygen saturation by pulse oximetry (80 - 85%). On general examination, he had clubbing of both fingers and toes with no cyanosis. Examination of the respiratory system revealed no abnormality. Heart sounds were normal, with no precordial or extraprecordial murmur or bruit. Review of other systems was normal. Routine blood examination showed normal total leukocyte count. His chest X-ray showed homogenous opacity with ill-defined margin in left lower zone surrounded by patchy areas of infiltrations. Arterial blood gas analysis in room air revealed hypoxemia (PaO_2_58 mm Hg, S_a_O_2_84%). He was treated conservatively with blood transfusion and intravenous fluid. Despite minimal chest-X-ray finding and stable hemodynamic status, his arterial saturation did not improve even with 50% oxygen inhalation for 30 min by venture mask (PaO_2_60 mm Hg, S_a_O_2_85%), which initiated further investigations. Doppler echocardiography was done to rule out intracardiac right-to-left shunt. Contrast enhanced CT scan of thorax showed a few aberrant vessels in left lower zone [Figure 2]. CT- pulmonary angiogram was done and it showed left lower lobe pulmonary arteriovenous malformation with a saccular aneurysm 35 ×30 mm size, mainly involving lateral and anterior basal segments with left lower pulmonary artery as feeding artery and left lower lobar pulmonary vein as the draining vein [Figure 3]. CECT scan of brain and spinal cord and CT scan of abdomen showed no abnormality. He was then referred to cardiothoracic surgeon who later successfully resected the aneurysm.

## DISCUSSION

PAVM is an uncommon disorder but may be an important differential diagnosis of common clinical problems such as hemoptysis, hypoxia, infiltration, mass lesion or pulmonary nodule in chest radiograph. Although majority of the cases are congenital, the average age at presentation attributable to the malformation is usually in the third or the forth decade, as in this case with massive hemoptysis. The clinical presentation of PAVM correlates best with the size[3] and the number[8] of shunts. Dyspnea is the most common respiratory complaint of PAVM, followed by hemoptysis.[5] Hemoptysis may rarely be fatal.[9] Life threatening hemoptysis may be caused by spontaneous rupture of dilated thin walls of the aneurysmal center of a PAVM.[9] Patients with large multiple shunts are hypoxemic and may experience dyspnea, clubbing, and polycythemia. However, dyspnea is sometimes markedly minimal compared to cyanosis and clubbing.[10]

PAVM can be classified as simple or complex type. Simple types (approx. 80%) have a single feeding artery and a single draining vein, whereas complex types (20%) have two or more of each.[1] Approximately 50-70% of PAVMs are located in the lower lobes that may be due to increased blood flow and pressure, and subsequent stretch due to hydrodynamic forces.[1] About 70% of patients have unilateral disease, 36% have multiple lesions.

The classic roentgenographic appearance of a PAVM is that of a round or oval mass of uniform density, frequently lobulated but sharply defined, more commonly in the lower lobes, and ranging from 1 to 5 cm in diameter.[5] PAVM may mimic infiltration, mass lesion, solitary or multiple nodule, and some rare conditions of pulmonary circulation[11] such as pulmonary varix, arterial aneurysm, and partial anomalous pulmonary venous return. However, in last three conditions there is no right-to-left shunt. The homogenous ill-defined opacity in our case that persisted even after repeated antibiotic courses and anti-tuberculosis treatment, with history of hemoptysis, was misinterpreted as tuberculoma. The ill-defined border may be due to associated parenchymal hemorrhage. The sensitivity of chest radiograph alone is 70% in diagnosing PAVM.

Various screening tests have been analyzed to date to efficiently identify the high-risk group with PAVM in patients with family history of HHT. However, no consensus has been developed regarding the best screening test. According to some authors, contrast echocardiography followed by shunt fraction assessment by 100% oxygen inhalation method is the best screening procedure,[5] while others suggest only contrast echocardiography in supine position to be the most sensitive (93%) test.[12] Shunt fraction can be most accurately assessed by measuring Pa _O2_and Sa _O2_after breathing 100% oxygen for 15 to 20 min by a mouthpiece connected by a one-way valve to oxygen source and a second valve allowing exhalation and the nasal passage closed by noseclip.[5] Venture masks and nonrebreather bag system are not acceptable because of potential leaks and entrainment of room air.[5] However, a working formula to assess shunt fraction >5% is Pa_O2_ <85 mm Hg or a Sa_O2_ <96% breathing room air.[5] However, this does not differentiate hypoxia due to V/Q mismatch. In our case, partial pressure of oxygen (PaO_2_) was 60 mm Hg and saturation (S_a_O_2_) 85% while breathing 50% oxygen by venture mask, which definitely indicates the presence of shunt fraction >5%. Now considering the clinical scenario, oxygenation status, and high degree of suspicion, contrast enhanced CT thorax and CT pulmonary angiography was advised. Pulmonary angiography and/or CECT is considered as the ‘gold standard’ test for the diagnosis of PAVM[12] as both have comparable sensitivity and specificity. Currently PA is indicated only while planning for embolization therapy.[12]

It is generally recommended that all symptomatic PAVMs and PAVM >2 cm diameter should be treated by surgery or embolotherapy.[5] White and co-workers[13,14] have recommended occlusion of all PAVMs with feeding artery ≥ 3 mm in diameter. Embolotherapy is preferable in most cases, which is beyond the scope of discussion in this article. However, different surgical procedures practiced for the same are ligation, local excision, segmentectomy, lobectomy, pneumonectomy, pulmonary artery resection by video-assisted thoracoscopy, and many others. The aneurysm in our case was resected successfully with relatively uneventful post-operative recovery phase.

To conclude, our patient had no history suggestive of any of the acquired causes of PAVM. Neither this is a case of HHT as per Curaæao criteria. Hence, most probably this is a case of congenital or idiopathic, single, simple type, pulmonary arteriovenous malformation in left lower lobe, manifested in an adult male with massive hemoptysis. In tuberculosis endemic country like India, sometimes there is a tendency among physicians to overdiagnose sputum negative PTB considering history of hemoptysis and ill-defined opacity in chest radiograph not resolving with antibiotic. However, detailed history taking, reviewing previous radiographs, and high degree of suspicion are of immense importance to diagnose a relatively rare disorder with very common presentation, else we will miss at least one case in every few thousands.
